# Coronal alignment increases ACL strain in vitro but lacks consistent clinical association with graft failure: A systematic review

**DOI:** 10.1002/jeo2.70765

**Published:** 2026-05-29

**Authors:** Joshua T. Bram, Tyler Khilnani, Mark F. Megerian, Alexander E. White, Akshay K. Raghuram, Anil S. Ranawat

**Affiliations:** ^1^ Hospital for Special Surgery Sports Medicine Institute New York New York USA

**Keywords:** ACL, coronal alignment, systematic review, valgus, varus

## Abstract

**Purpose:**

The purpose of this systematic review was to evaluate the existing literature on the impact of radiographic varus/valgus alignment on primary ACL rupture and graft failure.

**Methods:**

Three online databases (PubMed, Embase and Cochrane Library) were searched for the period of database inception until 31 July 2025. All clinical, biomechanical, or computational investigations assessing the influence of bony coronal plane alignment on primary ACL rupture/graft rupture were included. Study quality was assessed using the Methodological Index for Non‐Randomized Studies (MINORS) for clinical studies and Quality Appraisal for Cadaveric Studies (QUACS) scale for biomechanical studies.

**Results:**

Thirteen studies met inclusion criteria: four cadaveric (38 specimens) and nine clinical investigations. Cadaveric studies demonstrated a biomechanical disadvantage of mechanical axis deviation, with both excessive valgus and varus alignment increasing native ACL or ACL graft strain in vitro (11%–72% increase relative to neutral alignment). Up to 72% increased graft strain was observed with increased valgus alignment, suggesting valgus may pose greater biomechanical risk than varus. Conversely, clinical evidence for coronal malalignment as a risk factor for ACL rupture or ACL graft rupture was inconsistent. Some studies reported increased valgus alignment in ACL‐injured versus uninjured limbs, while others found no differences in either primary or revision ACL reconstruction setting. An association between greater varus alignment and inferior graft maturation was suggested.

**Conclusion:**

While biomechanical evidence indicates that both varus and valgus coronal malalignment increase ACL strain, the corresponding clinical evidence for its association with ACL rupture or ACL graft rupture remains limited. Based on the synthesis of data, preliminary thresholds of >5° valgus and >8° varus merit investigation as potential risk factors for ACL rupture/graft rupture. Given the lack of literature on this topic, further investigation is required to determine the effect of coronal malalignment on risk of ACL rupture and clinical thresholds that merit additional surgical intervention.

**Level of Evidence:**

Level IV.

## Graphical abstract

Coronal plane malalignment and ACL rupture risk. Systematic review—biomechanical vs. clinical evidence. Coronal alignment increases ACL strain biomechanically (up to 72% with valgus), but clinical evidence linking it to ACL rupture or graft failure remains limited and inconsistent.

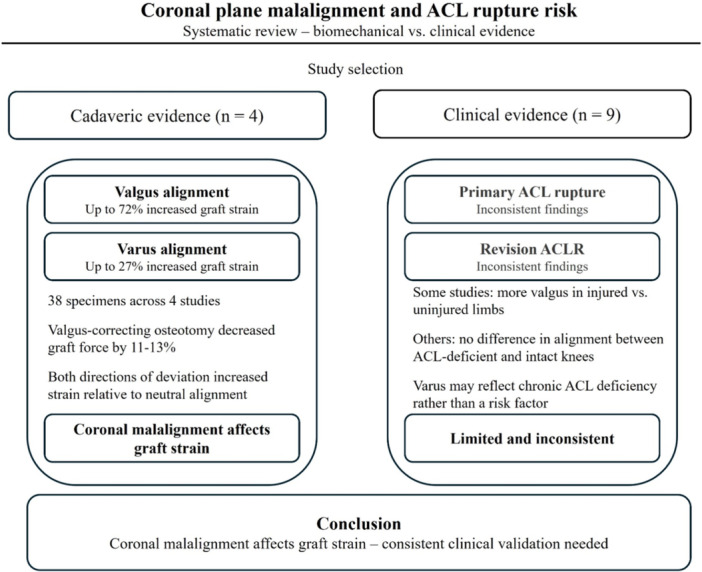

**Abbreviations**
ACL: anterior cruciate ligament
ACLR: anterior cruciate ligament reconstruction
BTB: bone‐tendon‐bone
HTO: high tibial osteotomy
MAD: mechanical axis deviation
MINORS: Methodological Index for Non‐Randomized Studies
mLDFA: medial lateral distal femoral angle
mMPTA: mechanical medial proximal tibial angle
MPTA: medial proximal tibial angle
MRI: magnetic resonance imaging
PRISMA: Preferred Reporting Items for Systematic Reviews and Meta‐Analyses
PSI: patient‐specific instrumentation
PTOA: posttraumatic osteoarthritis
PTS: posterior tibial slope
QUACS: Quality Appraisal for Cadaveric Studies
TFA: tibiofemoral angle
WBL: weight‐bearing line

## INTRODUCTION

Anterior cruciate ligament (ACL) ruptures are common but devastating injuries for athletes. Alignment parameters are often discussed in this setting, though there is a disparity in evidence between sagittal and coronal plane risk factors. Posterior tibial slope (PTS) has been extensively studied, with established thresholds showing that slopes >12° confer a significantly increased risk of ACL rupture or ACL graft rupture [7]. These findings have informed clear surgical indications and standardized treatment protocols to normalize alignment in the sagittal plane to decrease the risk of primary (or revision) ACL reconstruction failure. In contrast, comparable evidence‐based thresholds for coronal alignment have not been established despite coronal malalignment being frequently observed in ACL‐injured patients [4]. Among children with >2 years growth remaining, guided growth is commonly performed to address knee pain or instability—including in the setting of an ACL rupture or ACL graft rupture—and prevent the future development of degenerative arthritis [10, 20]. While a variety of thresholds for intervention in paediatric patients have been suggested, expert consensus would indicate guided growth in cases of mechanical axis deviation (MAD) > 10 mm or ≥ zone 2 of valgus [32]. High tibial or distal femoral osteotomies may be considered in select cases of revision ACLR with concurrent malalignment or medial compartment overload. However, clinical evidence in the literature to support this practice for isolated varus or valgus re‐alignment is limited [39].

Because ACL injuries frequently occur in the setting of combined dynamic valgus and internal rotation of the leg [9], and cadaveric studies have demonstrated that valgus loads significantly increase the force on the ACL, it stands to reason that static (i.e., osseous) coronal plane malalignment may contribute to risk for ACL rupture [1, 21].

The aim of this systematic review was to evaluate the available literature on the influence of radiographic varus or valgus alignment on primary ACL rupture and reconstruction graft failure. It was hypothesized that coronal plane malalignment would be associated with increased ACL strain and a greater risk of ACL rupture or graft failure.

## METHODS

**Figure 1:**
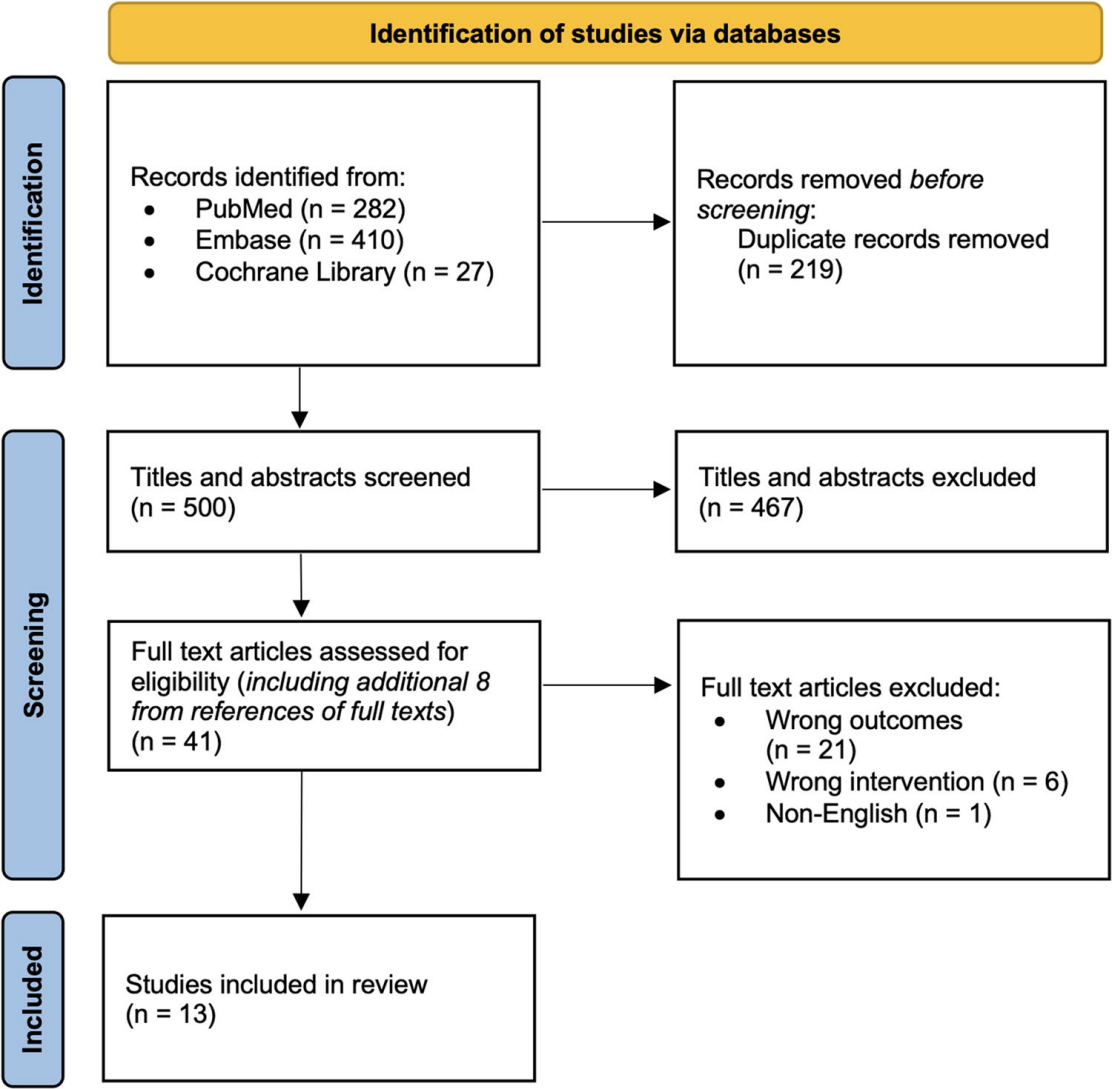
PRISMA diagram.

A systematic review was conducted in accordance with the Preferred Reporting Items for Systematic Reviews and Meta‐Analyses (PRISMA) guidelines (Figure 1). A comprehensive literature search was performed across three databases—PubMed, Embase, and the Cochrane Library—on 31 July 2025. A search strategy was designed to identify studies evaluating the association between radiographic coronal plane alignment and primary ACL rupture or ACL graft failure. Studies from database inception through 31 July 2025 were included. The complete search strategy is provided in Supporting Information S1: 1.

All clinical, biomechanical (cadaveric), or computational investigations that directly evaluated the influence of bony coronal plane alignment on primary ACL rupture or graft rupture/graft strain were considered for inclusion. Studies were excluded if they were published in a foreign language or evaluated dynamic varus/valgus instability (e.g., during the gait cycle or jumping) rather than bony (osseous) alignment. Additionally, clinical studies were further excluded if they used only clinical assessment of alignment (e.g., visual inspection rather than radiographic measurement), solely reported on other outcomes such as meniscal pathology or PTOA, focused on patients who underwent concomitant high tibial or distal femoral osteotomy, or studied alignment in the setting of a multi‐ligamentous knee injury (e.g., posterolateral corner or medial collateral ligament injury).

Clinical study quality and risk of bias were independently (J.B., T.K. and M.M.) assessed using the Methodological Index for Non‐Randomized Studies (MINORS) criteria (Table 1) [35], whereas the Quality Appraisal for Cadaveric Studies (QUACS) scale was used to assess the methodology of biomechanical articles (Table 2) [38]. In the MINORS criteria, each included study was scored across 12 domains for comparative studies or [8] domains for noncomparative designs, with scores ranging from 0 (not reported) to 2 (reported and adequate) per item. The QUACS scale includes 13 items, which are graded as either being 1 = yes/present or 0 = no/not stated. Across both scales, total scores were used to evaluate overall methodological quality.

**Table 1:** Methodological Items for Non‐Randomized Studies (MINORS) scores.

	Methodological items for nonrandomized studies								Additional criteria for comparative studies				
	A	B	C	D	E	F	G	H	I	J	K	L	Total score
Chung [3]	1	0	2	1	0	0	2	0	1	2	0	2	11
Kim [16]	1	1	2	2	1	2	2	0	‐	‐	‐	‐	11
Lazaro [19]	1	2	2	2	1	2	2	0	‐	‐	‐	‐	12
Mitchell [25]	2	1	2	2	2	0	2	0	2	2	1	2	18
Sato [33]	2	1	2	2	2	2	0	0	‐	‐	‐	‐	11
Sevim [34]	2	2	2	2	2	1	0	2	1	2	1	2	19
Thein [36]	2	2	2	1	2	0	0	0	‐	‐	‐	‐	9
Thompson [37]	2	2	2	2	2	0	0	2	‐	‐	‐	‐	12
Won [39]	2	1	2	2	2	0	0	2	2	1	1	2	17

*Note*: MINORS criteria according to Slim et al. [35], where 0 = not reported, 1 = reported but inadequate, and 2 = reported and adequate. The maximum overall scores are 16 and 24 for noncomparative and comparative studies, respectively, with higher scores representing better study quality.

Abbreviations: A, a clearly stated aim; B, inclusion of consecutive patients; C, prospective data collection; D, endpoints appropriate to the aim of the study; E, unbiased assessment of the study endpoint; F, follow‐up period appropriate to the aim of the study; G, loss to follow‐up <5%; H, prospective calculation of study size; I, an adequate control group; J, contemporary groups; K, baseline equivalence of groups; L, adequate statistical analyses.

**Table 2:** Quality Appraisal for Cadaveric Studies (QUACS) scale grades.

Author	A	C	B	D	E	F	G	H	I	J	K	L	M	Total
Gelhaus [11]	1	0	1	0	0	0	1	1	0	0	0	1	1	6
Imhoff [13]	1	1	1	1	1	0	1	1	1	1	1	1	1	12
Mehl [24]	1	1	1	1	0	0	1	1	1	1	1	1	1	11
Van de Pol [31]	1	1	1	1	0	0	1	1	1	1	1	1	1	11

*Note*: QUACS criteria according to Wilke et al. [38].

Abbreviations: A, objective stated; B, basic information about sample is included; C, applied methods are described comprehensibly; D, study reports condition of the examined specimens; E, education of dissecting researchers is stated; F, findings are observed by more than one researcher; G, results presented thoroughly and precise; H, statistical methods appropriate; I, details about consistency of findings are given; J, photographs of the observations are included; K, study is discussed within the context of the current evidence; L, clinical implications of the results are discussed; M, limitations of the study are addressed.

## RESULTS

The initial search yielded 719 articles, of which 219 duplicates were automatically removed using Covidence systematic review software (Veritas Health Innovation). The remaining 500 titles and abstracts were independently screened by two reviewers (J.T.B. and T.K.) for relevance using standardized methodology. After title/abstract screening, 41 articles were selected for full review. Of these, 13 studies merited inclusion, including two abstracts.

### Cadaveric/biomechanical

A total of four included articles were conducted in a cadaveric setting [11, 13, 24, 31] (Table 3). Gelhaus et al. simulated stepwise increases in varus alignment with a reconstructed ACL through performance of a lateral distal femoral osteotomy in [8] cadaveric specimens [11]. Significant increases in forces (up to 27% higher) experienced by the reconstructed ACL were observed at 8° of varus alignment compared to neutral axes, leading the authors to conclude that >5° varus alignment increases load on the ACL. Van de Pol similarly evaluated the influence of simulated varus alignment through both the middle of the medial compartment (~30% WBL, ~6.5° varus) and at the medial edge of the tibial plateau (100% WBL, ~12° varus) on native ACL tension [31]. Forces on the ACL were significantly higher at both a 50% and 100% varus WBL (54 and 38 N, respectively) compared to neutral alignment (31 N), which was consistent in both knee flexion and extension.

**Table 3:** Included studies and findings.

Author	Year	Journal	Setting	Findings
Chung [3]	2011	JOS	Clinical	No difference in TFA or mechanical axis between 28 ACL‐deficient and 20 ACL‐intact knees
Gelhaus [11]	2025	J ISAKOS	Cadaver	Increased strain on ACL at 30–40° knee flexion with 8° varus alignment compared to neutral alignment
Imhoff [13]	2021	AJSM	Cadaver	Isolated 5° valgus‐producing HTO reduced the forces on a reconstructed ACL graft by 12.7% and 11.4% at 200 and 400 N applied axial compression, respectively
Kim [16]	2011	CORR	Clinical	For patients with uncorrected baseline varus alignment undergoing primary ACLR, there were no differences in postoperative stability or functional scores at minimum 2‐year follow‐up for 201 knees based on degree of varus alignment
Lazaro [19]	2017	OJSM	Clinical	The injured limb demonstrated more valgus MAD (6.6 vs 1.4 mm) and mLDFA (85.2° vs. 86.1°) compared to the uninjured limb in patients with primary ACL tears
Mehl [24]	2020	KSSTA	Cadaver	ACL graft forces significantly increased at 85% and 115% valgus weight‐bearing line (96 and 110 N), respectively) compared to 50% (64 N) WBL (i.e., neutral alignment)
Mitchell [25]	2018	Arthroscopy	Clinical	No difference in coronal alignment (measured as mechanical axis deviation) between primary and revision ACLR groups
Sato [33]	2025	Arthroscopy	Clinical	Negative correlation between preoperative MPTA and postoperative ACL graft MRI relaxation times (T1⍴, T2 and UTE T2*) suggesting greater varus alignment associated with inferior graft maturation
Sevim [34]	2024	OJSM	Clinical	Patients with chronic ACL deficiency have greater medial proximal tibial angle (1.4° vs. 0.1°) compared to patients with acute ACL tears, while there was no difference in TFA or MPTA between the injured/uninjured limbs of the acute group
Thein [36]	2015	AJSM	Clinical	Across 74 patients with ACL tears, the injured leg was in significantly less varus (i.e., more valgus) compared to the uninjured leg (0.7° vs. 1.3°), which was more pronounced for patients with both medial and lateral meniscus tears (by 1.4°)
Thompson [37]	2025	AJSM	Clinical	Of 250 patients with primary ACL tear, 31% had varus alignment and 24% had valgus alignment, driven in the majority of patients by the MPTA (contributing to alignment 1.8‐times more frequently than the mLDFA)
Van de Pol [31]	2009	Arthroscopy	Cadaver	Tension on the ACL was significantly higher at a 50% and 100% simulated varus weight‐bearing line (54 and 38 N, respectively) compared to 0% (31 N) WBL (i.e., neutral alignment)
Won [39]	2013	CORR	Clinical	Compared to primary ACLR, patients undergoing revision ACLR more often had varus malalignment ≥5°(19% vs. 8%) and high‐grade medial meniscus injury (34% vs. 16%)

Abbreviations: AJSM, American Journal of Sports Medicine; CORR, Clinical Orthopaedics and Related Research®; J ISAKOS, Journal of the International Society of Arthroscopy, Knee Surgery and Orthopaedic Sports Medicine; JOS, Journal of Orthopaedic Surgery; KSSTA, Knee Surgery, Sports Traumatology, Arthroscopy; OJSM, Orthopaedic Journal of Sports Medicine.

Imhoff et al., as part of an investigation of anterior closing wedge osteotomy to address elevated PTS, performed an isolated 5° valgus‐producing osteotomy in 10 cadaveric knees with baseline varus deformity [13]. Correction of varus in the ACL‐reconstructed state led to a decrease in ACL graft force of 12.7% and 11.4% at 200 and 400 N of applied axial compression, respectively, suggesting corrective realignment osteotomies decrease graft strain and may reduce ACL graft failure. However, isolated valgus‐producing osteotomy led to an increase in anterior tibial translation in the ACL‐deficient knee and trended towards significance in the reconstructed state, leading the authors to conclude that isolated varus‐correcting high tibial osteotomy (HTO) in the setting of chronic ACL deficiency is inadequate. Starting from neutral alignment, Mehl et al. performed a valgus‐producing lateral distal femoral osteotomy in 10 cadaveric specimens with ACL reconstructions. They observed that ACL graft forces significantly increased at both an 85% (medium‐grade, mechanical axis through the lateral compartment) and 115% (high‐grade, mechanical axis lateral to the plateau) weight‐bearing line (96 and 110 N, respectively) compared to neutral alignment (64 N) [24].

### Clinical

Nine articles evaluated coronal alignment in a clinical setting [3, 16, 19, 25, 33, 34, 36, 37, 39] (Table 3). Coronal malalignment was commonly observed in primary ACL rupture, with Thompson et al. reporting that only 45% of patients demonstrate neutral alignment as defined as within 1 standard deviation of the mean [37]. Most frequently, changes in alignment originated in the proximal tibia (55% of varus and 65% of valgus), and female patients more often had baseline valgus alignment.

When assessing internal controls, Chung et al. observed no difference in MAD or tibiofemoral angle between 28 ACL‐deficient and 20 ACL‐intact knees as well as no difference between the injured and uninjured extremities for the ACL‐deficient group [3]. They concluded that the literature evidence is not strong enough to support an association between static varus/valgus alignment and noncontact ACL injuries. Conversely, Lazaro et al. reported that in a paediatric population, the ACL‐injured extremity was shown to have a higher, more often valgus MAD of 6.6 mm (vs. 1.4 mm in the uninjured leg), driven by the mLDFA [19]. Thein et al. similarly observed greater valgus alignment (0.7° varus vs. 1.3° varus) in tibiofemoral angle for the injured vs uninjured limb [36].

In 201 patients with baseline varus alignment—including 21% with ≥15 mm deviation—undergoing primary ACLR, Kim et al. reported no differences in objective stability or patient‐reported outcomes at 2 years postoperative based on the degree of MAD [16]. They strongly concluded that stability and functional scores after ACLR are not adversely affected by primary varus alignment and there is no indication for HTO in this setting. However, a postoperative MRI analysis of knees 2 years after primary ACL observed a negative correlation between preoperative mMPTA and postoperative ACL graft MRI relaxation times (T1⍴, T2 and UTE T2*) [33]. This indicates that greater varus alignment may be associated with inferior graft maturation. In patients with more chronic ACL injury as compared to acute tears, there may be decreased mMPTA and greater tibiofemoral angle (i.e., more varus) [34].

In the revision ACL setting, the literature was similarly inconsistent. Mitchell et al. observed no difference in MAD between skeletally mature patients undergoing primary versus revision ACLR [25]. Conversely, Won et al. reported a greater proportion of patients with varus malalignment ≥5° in 58 patients undergoing revision ACLR vs 116 individuals undergoing primary ACLR (19% vs 8%) [39]. They posited that a greater number of patients requiring revision ACLR may benefit from a HTO to address underlying malalignment as well as an observed higher proportion of high‐grade medial tibiofemoral osteoarthritis.

## DISCUSSION

The most important finding of the present study is that while there is a clear biomechanical disadvantage of excessive varus or valgus on ACL strain at time‐zero, the corresponding clinical evidence for coronal malalignment as a risk factor for primary ACL rupture or graft failure is limited. In cadaveric studies, greater ACL strain was observed with valgus alignment compared to varus alignment. Based on synthesis of available data, valgus deviation appears to be a stronger risk factor for ACL rupture or ACL graft rupture, and preliminary thresholds of >5° valgus and >8° varus, derived primarily from cadaveric studies with modest quality scores (QUACS 6‐12/13), merit consideration as potential indications for alignment correcting osteotomies, though prospective validation is needed. Additionally, given the observation of proximal tibial varus in patients with chronic (>5 years) versus acute ACL deficiency [34], it is unclear whether varus malalignment represents a true risk factor for ACL rupture/graft rupture, or a chronic sequela of unaddressed ACL deficiency leading to progressive medial compartment degeneration.

Several cadaveric studies demonstrated the biomechanical influence of coronal malalignment on ACL kinematics. As demonstrated by Mehl et al., a lateral distal femoral osteotomy produced the largest increases in ACL graft strain compared to neutral alignment [24]. Conversely, Van de Pol et al. [31] and Gelhaus et al. [11] both demonstrated more modest increases in ACL strain at >6.5° and >5° of varus, respectively, as compared to neutral. Furthermore, Imhoff et al. demonstrated that varus‐correcting osteotomies towards neutrality decreased ACL strain by 11%–13% [13]. The results of these studies suggest there is increased tension on the native or reconstructed ACL regardless of the direction of deviation, highlighting the biomechanical advantage of a neutral mechanical axis. Greater valgus deviation produced the highest tension on the ACL and thus supports valgus as a more significant risk factor. While intuitively this follows given that a dynamic valgus moment at the knee is a common mechanism for ACL rupture, further investigation linking bony valgus malalignment to true dynamic valgus is needed.

However, these in vitro findings did not translate to the few published clinical studies in a consistent or reliable manner. Two studies reported that ACL‐deficient knees were more likely in valgus as compared to the contralateral ACL‐intact knee [19, 36]. While statistically different, the clinical relevance of a 0.6° difference in coronal alignment according to Thein is questionable. Furthermore, other studies directly contradicted these radiographic findings, demonstrating no difference in coronal alignment between ACL‐deficient and ACL‐intact knees [3, 16]. In the revision ACLR setting, the influence of coronal malalignment was also inconsistent. Won et al. reported a higher rate of >5° varus malalignment and medial tibiofemoral degeneration in those undergoing revision ACLR [39], while Mitchell et al. observed no difference in coronal alignment between primary ACLR patients and a matched group that underwent revision ACLR [25]. These inconsistent clinical results suggest that mild malalignment may be tolerated, but specific thresholds may demarcate increased risk. Conversely, guided growth procedures are routinely performed for valgus deformity in paediatric populations, typically when alignment exceeds 5° valgus [10]. This established practice suggests clinical recognition of excessive valgus as a modifiable risk factor for future knee pathology through mechanical overload and progressive joint degeneration, including increased risk of ACL rupture or ACL graft rupture. The widespread adoption of these preventive interventions, despite limited quantitative data, underscores both the perceived importance of coronal alignment and the need for definitive prospective studies to establish evidence‐based thresholds.

Other studies commonly report that coronal malalignment subjectively contributes to ACL rupture or re‐tear. In the largest evaluation of revision ACLR cases, the MARS group attributed post‐operative failure after primary ACLR to malalignment in 4% of patients [22]. Noyes et al. similarly published three studies specifically focusing on revision ACLR. In 66 knees undergoing revision ACLR with BTB allograft, they noted that eight (12%) were indicated for an HTO for unaddressed varus malalignment based on clinical judgment rather than standardized alignment criteria, though did not report those specific outcomes [29]. In another evaluation of 21 patients undergoing ACLR with quad tendon autograft, they posited that previously untreated varus malalignment contributed to re‐tear in 25% of cases. Outcomes after combined HTO/revision ACLR were again not reported [27]. In a third series of 54 knees, nine (16%) required an HTO for unaddressed varus malalignment (6.5° average) [28]. While these studies demonstrate that a reasonable proportion of patients undergoing revision ACLR may have coronal malalignment, they are limited by lack of objective radiographic measurement of coronal alignment. Notably, patients who underwent concomitant HTO had inferior outcomes compared to those who underwent revision ACLR alone [28]. This finding may reflect the greater severity of malalignment and articular degeneration in the HTO subgroup rather than a detrimental effect on the osteotomy itself; however, the absence of quantified pre‐operative alignment data precludes definitive interpretation.

Another concern when evaluating research reporting on coronal alignment in the setting of ACL rupture or ACL graft rupture is the chronicity of tear and association with medial tibiofemoral PTOA. It is not clear if increased varus alignment either causes and/or represents sequelae of ACL tear. While Sevim et al. observed greater proximal tibial and tibiofemoral varus for patients with chronic ACL rupture of >5 years duration compared to acute ACL rupture, this could have been secondary to the natural history of ACL insufficiency [34]. Chronic ACL deficiency is a well‐established risk factor in progressive medial compartment PTOA, which in turn leads to joint space narrowing and secondary varus over time. Given the orientation of the ACL from its origin on the lateral intercondylar notch and insertion on the tibial spines, it is well‐established that medial compartment PTOA is a long‐term outcome in the setting of ACL deficiency. Depending on the timing of imaging and reconstruction, it is likely that observed varus alignment in some clinical studies may be a result‐of rather than causative‐of ACL rupture. Caution is thus urged when suggesting that varus alone is a risk factor for primary ACL rupture given the inconclusive literature [23].

It is important to review that osseous malalignment in the coronal plane can originate from either the proximal tibia or the distal femur and is routinely corrected at the site of deformity with either a HTO or DFO, particularly in the revision setting. In the sagittal plane, anterior closing wedge HTO has been effective in reducing PTS, a known risk factor for increased anterior tibial translation and ultimately primary/revision ACL rupture [12, 26]. Sagittal plane corrections have strong evidence basis, with PTS reduction showing clear benefits when slopes exceed 12°. In the coronal plane, HTO has been utilized largely in the revision ACLR setting to correct varus and/or to offload the medial compartment in those with symptomatic PTOA [14]. However, coronal plane HTO lacks similar evidence‐based thresholds, being performed primarily for empirical reasons rather than defined alignment criteria. Several systematic reviews have holistically evaluated combined ACLR and HTO, and generally report improved alignment, functional scores, and knee stability [5, 17]. However, there is inconsistent reporting of indications (i.e., stability versus arthritis), injury factors (i.e., acute versus chronic tear), surgical history (i.e., primary versus revision), and surgical technique (i.e., 1‐ or 2‐stage, closing versus opening wedge osteotomy, ACL graft choice). Furthermore, HTO is a technically demanding procedure that may carry unintended morbidity. Truly isolated coronal or sagittal correction is difficult to achieve [2]. Medial opening wedge HTO techniques, for example, can result in an unintended 2.1–2.9° change in PTS [18]. While modern innovations such as patient‐specific instrumentation (PSI) have been shown to reduce the rate of unintended multiplanar deviations during HTO, such technology is not readily available and requires specific training [6]. Additionally, there are currently no clinical studies that directly compare combined HTO/ACLR to isolated ACLR when addressing coronal malalignment. Given the technical demands of the procedure, surgeons should be cautious when indicating HTO along with ACLR. Currently, the most established indication for realignment osteotomy with concurrent ACLR is symptomatic arthritis, with the goal of offloading the affected compartment and reducing the progression of arthrosis, rather than decrease risk of ACL re‐rupture. The preliminary thresholds of >5° valgus and >8° varus proposed herein are derived primarily from cadaveric biomechanical data and underscore the need for prospective clinical validation.

While beyond the scope of this review, there is myriad data demonstrating an association between coronal malalignment and concomitant injury to other soft tissue structures around the ACL. Okoroha et al. reported that patients with tibia vara angles >3° had 5.2‐fold higher odds of concomitant lateral meniscus posterior root tears in context of ACL injury [30]. This relative risk quantification for associated injuries contrasts with the absence of similar data for ACL injury itself. Varus alignment has also been associated with medial meniscal injury. Kawashima et al. noted that patients with varus alignment undergoing early ACLR (<60 days) demonstrated 7‐fold higher odds of medial meniscus injury compared to a delayed group, though interestingly found 4‐fold lower odds of lateral meniscus injury at the time of ACLR [15]. Last, strict exclusion criteria were applied including removal of the numerous articles on the impact of dynamic valgus on ACL rupture or ACL graft rupture. While it is appreciated that this is a common noncontact ACL rupture mechanism, the soft tissue tension of other stabilizers about the knee may be as if not more important than osseous parameters. The present review therefore attempted to isolate the influence of solely coronal malalignment.

There are several limitations to this systematic review. The literature associating coronal malalignment with ACL rupture or ACL graft rupture is sparse and of largely low‐quality, making any summarization of the available data subject to potential bias. The included clinical studies varied in patient populations (e.g., skeletally immature vs. adult, primary vs. revision ACLR), alignment measurement techniques, and outcome definitions, which limits the ability to draw definitive conclusions. Furthermore, many of the clinical studies did not perform adequate adjustment for possible confounding factors such as sex, BMI, and concomitant injuries, which may influence the observed association between coronal alignment and ACL outcomes. Nevertheless, given the lack of meaningful research in this area, it was felt necessary to include all published data on this topic to identify a possible gap in literature. This association, along with the impact of any potential confounders, should certainly be the focus of future research. A stratified analysis separating paediatric from adult populations was not feasible given the limited number of included studies. Based on biomechanical data showing comparatively increased strain under valgus over varus alignment, valgus alignment appears to pose greater risk than varus, though prospective validation is required.

## CONCLUSION

While biomechanical evidence indicates that both varus/valgus coronal malalignment increase ACL strain, the corresponding clinical evidence for its association with ACL rupture or ACL graft rupture remains limited. Based on the synthesis of available data, thresholds of >5° valgus and >8° varus merit investigation as potential risk factors for ACL rupture or ACL graft rupture. However, further research is needed to identify whether static valgus alignment predisposes to ACL rupture/graft rupture and whether varus malalignment causes or is a consequence of chronic ACL deficiency.

## FUNDING INFORMATION

The authors have no funding to report.

## AUTHOR CONTRIBUTIONS

Joshua T. Bram, Alexander E. White and Anil S. Ranawat were involved in conceptualization. Joshua T. Bram, Tyler Khilnani and Mark F. Megerian were involved in investigation and methodology. Akshay K. Raghuram is involved in project administration and resources. Joshua T. Bram, Tyler Khilnani, Mark F. Megerian and Akshay K. Raghuram were involved in writing—original draft preparation. Alexander E. White and Anil S. Ranawat were involved in writing—reviewing and editing.

## CONFLICT OF INTEREST STATEMENT

The authors declare no conflicts of interest.

## ETHICS STATEMENT

The authors have nothing to report.

## Supporting information

Varus_Valgus_ACL_SR_supplement1_11182025.

## Data Availability

The datasets generated and analyzed during this study are available from the corresponding author on request. All data underlying the findings are derived from published studies that are publicly available and fully cited within this article.
