# Supplementary material for: Coronal alignment increases ACL strain in vitro but lacks consistent clinical association with graft failure: A systematic review
Source: J Exp Orthop. 2026 May 29;13(2):e70765. doi: 10.1002/jeo2.70765 (PMC13239122; doi:10.1002/jeo2.70765)
Supplement: Supplementary file 1 — Varus_Valgus_ACL_SR_supplement1_11182025. [file JEO2-13-e70765-s001.docx]

**Supplement 1.** Search criteria

- PubMed (282)
  - ("Anterior Cruciate Ligament"[MeSH] OR “Anterior Cruciate Ligament Reconstruction”[MeSH] OR “Anterior Cruciate Ligament Injuries”[MeSH] OR "ACL"[tiab]) AND ( "Genu Varum"[MeSH] OR "Genu Valgum"[MeSH] OR “Varus”[tiab] OR “Valgus”[tiab] OR “Coronal alignment”[tiab] OR “Mechanical axis”[tiab]) AND (“Rupture”[MeSH] OR “Rupture”[tiab] OR “Re-rupture”[tiab] OR “Rerupture”[tiab] OR “Tear”[tiab] OR “Re-tear”[tiab] OR “Retear”[tiab])
- Embase (410)
  - ('anterior cruciate ligament'/exp OR 'anterior cruciate ligament injury'/exp OR 'anterior cruciate ligament rupture'/exp OR 'anterior cruciate ligament reconstruction'/exp OR 'acl':ti,ab,kw OR 'acl reconstruction':ti,ab) AND (‘varus deformity’/exp OR ‘valgus deformity’/exp OR ‘varus’:ti,ab,kw OR ‘valgus’:ti,ab,kw OR ‘coronal alignment’/exp OR ‘coronal alignment’:ti,ab,kw OR ‘mechanical axis deviation’/exp OR ‘mechanical axis’:ti,ab,kw) AND (‘rupture’:ti,ab,kw OR ‘re-rupture’:ti,ab,kw OR ‘rerupture’:ti,ab,kw OR ‘tear’:ti,ab,kw OR ‘re-tear’:ti,ab,kw OR ‘retear’:ti,ab,kw)
- Cochrane library (27)
  - ("anterior cruciate ligament"[MeSH] OR "anterior cruciate ligament reconstruction"[MeSH] OR "anterior cruciate ligament injuries"[MeSH] OR "acl"ti,ab,kw OR “acl reconstruction”ti,ab,kw) AND (“genu varum”[MeSH] OR “genu valgum”[MeSH] OR “varus”ti,ab,kw OR “valgus”ti,ab,kw OR “coronal alignment”ti,ab,kw OR “mechanical axis”ti,ab,kw) AND (“Rupture”[MeSH] OR “Rupture”ti,ab,kw OR “Re-rupture”ti,ab,kw OR “Rerupture”ti,ab,kw OR “tear”ti,ab,kw OR “re-tear”ti,ab,kw OR “retear”ti,ab,kw)
